# Juvenile hormone regulates the shift from migrants to residents in adult oriental armyworm, *Mythimna separata*

**DOI:** 10.1038/s41598-020-66973-z

**Published:** 2020-07-15

**Authors:** Lei Zhang, Lili Cheng, Jason W. Chapman, Thomas W. Sappington, Juanjuan Liu, Yunxia Cheng, Xingfu Jiang

**Affiliations:** 1 State Key Laboratory for Biology of Plant Diseases and Insect Pests, Institute of Plant Protection, Chinese Academy of Agricultural Sciences, Beijing, 100193 China; 2 Centre for Ecology and Conservation, and Environment and Sustainability Institute, University of Exeter, Penryn Cornwall, TR10 9FE United Kingdom; 3 Department of Entomology, Nanjing Agricultural University, Nanjing, China; 4 USDA-ARS Corn Insects & Crop Genetics Research Unit, Genetics Laboratory, Iowa State University, Ames, IA 50011 USA

**Keywords:** Ecology, Animal migration

## Abstract

In migratory insects, increasing evidence has demonstrated juvenile hormone (JH) is involved in regulating adult reproduction and flight. Our previous study demonstrated that the switch from migrants to residents in *Mythimna separata* could be induced by adverse environmental conditions during a sensitive period in adulthood (the first day post-emergence), but the role of JH in this switch is not clear. Here, we found a significantly different pattern of JH titers between migrants and residents, with migrants showing a slower release of JH during adulthood than residents. Application of JH analogue (JHA) in the 1-day-old adults, significantly accelerated adult reproduction and suppressed flight capacity. The pre-oviposition period and period of first oviposition of migrants treated with JHA were significantly shorter, while the total lifetime fecundity and mating percentage increased. The flight capacity and dorso-longitudinal muscle size of the migrants were decreased significantly when treated with JHA. The effect of JHA on reproduction and flight capacity indicate that JH titers during the sensitive period (first day post-emergence) regulates the shift from migrants to residents in *M. separata*.

## Introduction

Insect migration is an important behavioral strategy, which allows species to adapt to environmental variations and seek new habitats^1^. However, in many migratory species, not all individuals within a population are destined to develop into migrants, and often both migrant and resident morphs coexist. In crickets, aphids and planthoppers, the wing form (long or short/absent) is often affected by changes in the environmental conditions experienced during development^2–4^. In such species, migrants show stronger flight capacity and poorer reproductive capacity than residents. In migratory Endopterygota, such as Coleoptera^5^ and Lepidoptera^6–8^, the migrant phenotype is characterized by the longer flight duration and delayed reproduction compared to the resident phenotype, but there are no external morphological differences between migrants and residents. Therefore, flight capacity, and the length of the pre-oviposition period (POP) can be used to distinguish migrants from residents. Furthermore, the period of first oviposition (PFO) measuring synchrony of first egg-laying by cohorts of post-migratory females, indicates the time-window for onset of oviposition after migration; the greater synchrony of egg-laying (i.e. a shorter PFO) leads to an increase in egg and subsequent larval densities^9,10^.

It has been frequently hypothesized that elevated levels of juvenile hormone (JH) during a sensitive period of development completely or partially blocks the development of wings and flight muscles^4–8,11–14^. Many studies have demonstrated sensitive periods during development, when JH or a JH analogue (JHA) impacts on wing development and the “Oogenesis-flight syndrome”^15–19^. Besides the influence on development of immature stages, an increase of JH during the early adult stage is typically believed to accelerate ovarian growth in flightless morphs^11,12,19–21^. Lower JH titers normally stimulate flight in the pre-reproductive adult, whereas higher titers are required to complete ovarian development^22^. For instance, in wing-polymorphic crickets, the application of methoprene (JHA) increased the ovarian growth, egg production and incidence of flight muscle histolysis in long-winged adult crickets^19^.

The oriental armyworm, *Mythimna separata* (Walker) (Lepidoptera: Noctuidae), is a major migratory pest of grain crops in China and other Asian countries. Larvae that experienced poor nutrition, high larval density, a short photoperiod, and low temperature tend to develop into migrants^23–26^. Similar to wing-dimorphic species, migrants have a stronger flight capacity coupled with a longer POP than residents^23,24,27–32^, although there are no morphological differences between them. Interestingly, we previously found that *M. separata* larvae which experienced environmental conditions conducive to develop into migrants, would shift back to residents if the adults encountered a lack of nectar, low temperature or a long photoperiod conditions on the first day post-emergence^30–32^. Such a switch from migrants to residents leads to a significantly curtailed POP and flight capacity. Because most previous studies have focused on the sensitive period during larval development, here we investigated the role of JH in determining migratory potential in the adult stage of *M. separata*^31,32^.

JH plays a pivotal role in regulating reproduction and migration in *M. separata*, in which there are two types of JH (JH I and JH II)^31,33^. JH levels are low before migration, when moths are sexually immature and do not mate, whereas the level increases significantly in females after migratory flight is commenced, and is associated with termination of migratory behavior and the switch to reproduction^33,34^. Flight activity of *M. separata* significantly influences JH biosynthesis rates by the corpora allata (CA), depending on the age at which the moths fly, and 1–3 days after adult emergence seems to be the critical period for activation of the CA^35^. Jiang and Luo^33^ suggested that lower JH titers induced adult migration, whereas higher titers stimulated oogenesis. Direct evidence of JH regulating migration of *M. separata* was that the application of JHA on day 1 after eclosion, significantly reduced the adult flight capacity and energy content after treatment^36^. Adult starvation may activate JH synthesis, accelerate ovarian development and fight muscle degradation, and consequently a shift from migrant to resident phenotype. An early peak of hemolymph JH titers and high expression of the allatotropin (AT) gene in the adult seem to be the main factor contributing to the shifting of migrants to residents^30,32^. However, direct evidence of the role of JH in driving the shift from migrants to residents induced by environmental factors is lacking. In this study, we hypothesized that JH titers in a specific sensitive period regulated the shifting of migrants to residents, and higher JH level accelerated ovarian development and oviposition, and induced the degradation of flight muscle and decline of flight capacity. Therefore, differences in JH titers between migrants and residents, and the direct influence of JHA on flight parameters, were studied.

## Results

### Different expression patterns of JH between migrants and residents

**Figure 1:**
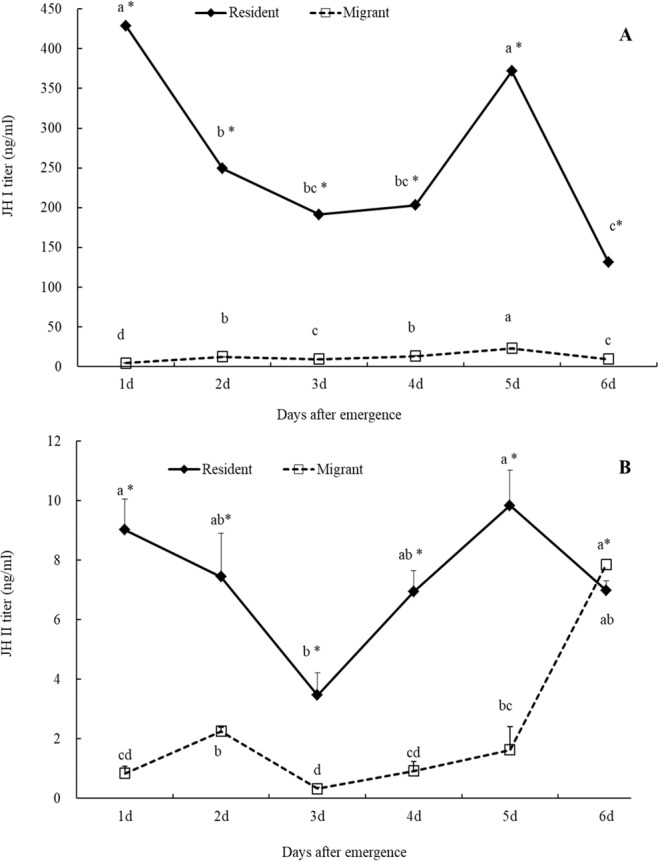
The JH I (**A**) and JH II (**B**) titers of migrant and resident adults in *Mythimna separata*. The asterisk indicates a significant difference between migrants and residents at the 5% level by Student’s test. The same letters mean no significant differences among female ages at the 5% level by Tukey’s HSD test.

There were significant differences in JH titers (JH I and JH II) between the resident and migrant female adults at the same age (Fig. 1, *P* < 0.05). JH I titer differed significantly with age, morph and the interaction between the two (Age: *F*_*5, 24*_ = 47.85; *P* < 0.0001; Morph: *F*_*1, 24*_ = 1358.24, *P* < 0.0001; Age*Morph: *F*_*5, 24*_ = 46.74, *P* < 0.0001, Table 1). JH I titers in the residents were significantly higher than those in the migrants on every one of the first 6 days after emergence (day 1: *t*_*1, 4*_ = 13.24, *P* < 0.01; day 2: *t*_*1, 4*_ = 30.83, *P* < 0.01; day 3: *t*_*1, 4*_ = 21.57, *P* < 0.01; day 4: *t*_*1, 4*_ = 17.14, *P* < 0.01; day 5: *t*_*1, 4*_ = 19.47, *P* < 0.01; and day 6: *t*_*1, 4*_ = 15.11, *P* < 0.01; Fig. 1A). In residents, the highest JH I peak appeared on day 1 after adult emergence and then decreased significantly on day 2 (*F*_*5,12*_ = 47.19, *P* < 0.05, Fig. 1A); then, it steadily increased from day 3 and reached a significantly higher peak on day 5 after emergence (*F*_*5,12*_ = 47.19, *P* < 0.05). After day 5, the JH I titer sharply decreased again and was significantly lower than those of days 1, 2 and 5 (*F*_*5, 12*_ = 47.19, *P* < 0.05). In migrants, however, the JH I titer on day 1 was the lowest and significantly lower than those of day 2 to day 6 (*F*_*5, 12*_ = 237.99, *P* < 0.05, Fig. 1A); peak levels occurred on day 5 after adult emergence which was clearly higher than those of other days (*F*_*5, 12*_ = 237.99, *P* < 0.05).

**Table 1:** Two-way ANOVA analysis on JHI and JHII titer of adult *Mythimna separata* as a function of adult age and morph.

Source of variation	*df*	JH I		JH II	
		*F*	*P*	*F*	*P*
Age	5	47.85	<0.0001	13.64	<0.0001
Morph	1	1358.24	<0.0001	149.87	<0.0001
Age* Morph	5	46.74	<0.0001	11.95	<0.0001
Error	24				
Total variation	36				

There were also significant differences in JH II titers between different morphs (migrant and residents) and adult ages (Age: *F*_*5, 24*_ = 13.64; *P* < 0.0001; Morph: *F*_*1, 24*_ = 149.87, *P* < 0.0001; Age*Morph: *F*_*24*_ = 11.95, *P* < 0.0001; Table 1). JH II titers in residents were significantly higher than those of the migrants on days 1 to 5 (day 1: *t*_*1, 4*_ = 7.98, *P* < 0.01; day 2: *t*_*1, 4*_ = 3.55, *P* = 0.02; day 3: *t*_*1, 4*_ = 4.25, *P* = 0.01; day 4: *t*_*1, 4*_ = 8.36, *P* < 0.01; and day 5: *t*_*1, 4*_ = 6.59, *P* < 0.01; Fig. 1B), but not different on day 6 (*t*_*1, 4*_ = -2.22, *P* > 0.05, Fig. 1B). In residents, JH II titers significantly decreased from day 1 to day 3, and then sharply increased, peaking on day 5 (*F*_*5,12*_ = 5.12, *P* < 0.05, Fig. 1B). However, in migrants, the JH II titer on day 1 after emergence was significantly lower than that on day 2 or day 6 (*F*_*5,12*_ = 181.08, *P* < 0.05; Fig. 1B). Although the JH II titer declined after day 2, it increased again after day 3 and peaked on day 6 after emergence. Interestingly, JH II titers in residents peaked one day earlier than that in migrants.

### Dose-dependent effects of JHA on adult reproduction in migrants

In all doses of JHA treatment, topical application of JHA on migrant adults on day 1 post-emergence (1-day-old) had no effect on adult survival. No adults died within the first two days, and survival rates of adults in all JHA treatments did not significantly differ from the control. However, the JHA treatment significantly affected adult’s reproduction (Table 2). POP was significantly affected by different doses of JHA treatment (*F*_5,167_ = 19.01, *P* < 0.0001), with a significant reduction of the POP at concentrations of 6 and 60 μg/μL JHA, resulting in females under these treatments beginning to oviposit significantly earlier than the controls or those treated with lower doses (0.6, 0.06 and 0.006 μg/μL, Table 2). The PFO was also significantly negatively affected by the JHA concentration (*F*_5, 167_ = 6.01, *P* < 0.001). On average, the PFO of adults treated with 6 and 60 ug/uL JHA was 0.89 and 0.85 day, respectively, which was significantly shorter than that of the controls and JHA treatments using lower doses (Table 2). The mean PFO of moths treated with 0.006, 0.06, and 0.6 ug/uL JHA were not significantly different from those of controls at day 1 after emergence. Effects of JHA on lifetime fecundity were significantly dose-dependent (*F*_5, 167_ = 8.59, *P* < 0.001). Females treated with 0.6 and 6 ug/uL JHA produced more eggs than females from controls or JHA treatments with the lowest (0.006ug/uL) and the highest (60ug/uL) doses (Table 2). The oviposition periods were also affected by JHA (Table 2), and the 60ug/uL JHA treatment significantly shortened the oviposition period compared to the 0.006–6 ug/uL treatments (*F*_5,167_ = 6.84, *P* < 0.001), whereas it did not differ from the control (Table 2).

**Table 2:** Reproductive performance of *Mythimna separata* females treated by different doses of methoprene (JHA) on day 1 after emergence (mean ± SE).

JHA concentration (μg/μL)	Pre-oviposition period /day	Lifetime fecundity/eggs per female	Period of first oviposition / day	Oviposition period /day	Mating percentage (%)
0 (CK)	5.15 ± 0.18 ab	807.80 ± 68.94 b	2.15 ± 0.18 a	5.10 ± 0.45 ab	68.97 b
0.006	5.78 ± 0.30 a	849.00 ± 55.22 b	1.78 ± 0.30 a	6.25 ± 0.38 a	96.97 a
0.06	5.44 ± 0.27 a	908.59 ± 58.50 ab	1.44 ± 0.27 ab	6.00 ± 0.33 a	90.00 ab
0.6	4.29 ± 0.12 bc	1143.42 ± 54.23 a	1.29 ± 0.12 ab	6.68 ± 0.35 a	93.94 ab
6	3.89 ± 0.09 c	1121.03 ± 55.62 a	0.89 ± 0.09 b	5.50 ± 0.45 a	92.31 ab
60	3.85 ± 0.09 c	696.78 ± 79.21 b	0.85 ± 0.08 b	4.09 ± 0.20 b	81.82 ab

Means with the same letter in the same column are not significantly different at the 5% level by protected HSD test. Differences in mating percentage among the control (CK) and all experimental treatments were examined by a Chi-square test with the significance *P* = 0.05/15 = 0.0033.

JHA treatments significantly increased the mating percentage of adults, especially in the dose of 0.006ug/uL JHA (*χ*^*2*^ = 15.35, *df* = 5, *P* < 0.01, Table 2). But the significant difference was only found between treatment of 0.006 μg/μL dose of JHA by the partitions of Chi-square method (*χ*^*2*^ = 8.95, *df* = 1, *P* < 0.0033). No significant differences were found in other treatments and the control (*P* > 0.0033). The JHA treatment of 6 μg/μL resulted in the shortest POP, the highest lifetime fecundity, and the highest mating percentage (Table 2), suggesting that it should be selected as the test concentration in the following experiments to determine the effects on shifting migrants to residents.

### Determination of the sensitive period of adults to JHA

**Figure 2:**
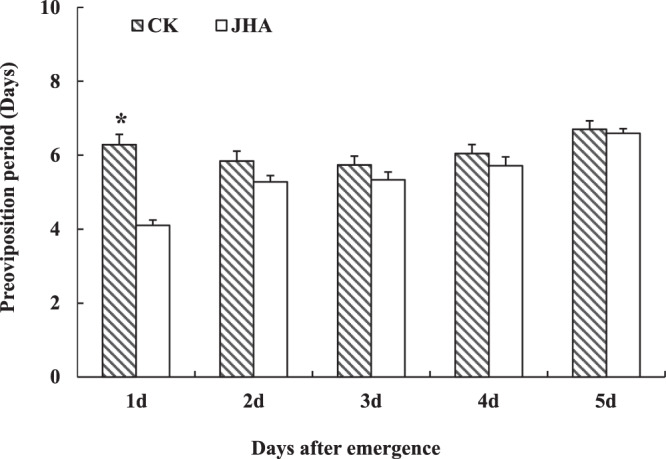
The pre-oviposition period (day) of migrant *Mythimna separata* females treated by JHA on 1–5 days after emergence (mean ± SE). * indicates significant differences between JHA treatment and control at *P* < 0.05 by Student’s t-test.

**Figure 3:**
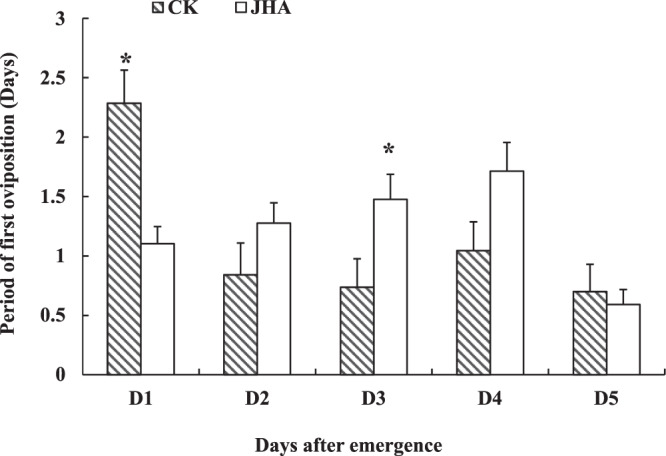
The period of first oviposition (day) of migrant *Mythimna separata* females treated with JHA on 1–5 days after emergence (mean ± SE). * indicates significant differences between the JHA treatment and control (CK) at *P* < 0.05 by Student’s t-test.

JHA (6ug/uL) treatment on the 1-day-old females significantly increased their mating percentage (*χ*^2^ = 5.28, *df* = 1, *P* = 0.02) and lifetime fecundity (*t*_1, 48_ = 2.36, *P* = 0.02), but this treatment on the 2 to 5-day-old females did not affect these adults reproduction. This JHA treatment on 1 to 5-day-old females did not affect the oviposition period of these females (Table 3). Similarly, the JHA treatment on the 1-day-old females significantly shorted the POP of adults (*t*_*1, 48*_ = -7.54, *P* < 0.01), but it did not affect the POPs when treated on the 2 to 5-day-old females (Fig. 2). Moths treated with JHA on the first day after emergence showed a shorter PFO than the moths without JHA (*t*_*1,48*_ = 4.08, *P* < 0.001), but when the JHA treatment on the 2 to 5-day-old females, the PFOs of these moths became longer than or equivalent to the control without JHA (Fig. 3).

**Table 3:** Reproductive performance of *Mythimna separata* females treated with 6 ug/uL Methoprene (JHA) in the first to fifth day after emergence (mean ± SE).

Reproductive parameter	Treatment	Female age when treated by JHA				
		1-day-old	2-day-old	3-day-old	4-day-old	5-day-old
Lifetime fecundity / eggs per female	CK	474.86 ± 88.66	900.53 ± 63.56	644.11 ± 51.45	599.41 ± 89.41	681.75 ± 59.32
	JHA	727.03 ± 64.67*	718.07 ± 65.96	607.38 ± 74.89	576.19 ± 62.36	776.59 ± 53.46
Oviposition period /day	Control	4.76 ± 0.58	7.95 ± 0.69	6.68 ± 0.44	7.05 ± 0.69	5.40 ± 0.44
	JHA	5.72 ± 0.37	6.52 ± 0.37	5.71 ± 0.29	6.43 ± 0.38	5.18 ± 0.35
Mating percentage /%	Control	63.64	70.37	64.29	62.86	83.33
	JHA	87.88*	74.36	72.41	70.00	81.48

Asterisk indicates significant difference between the control (CK) and JHA treatment at the 5% level by Student’s t-test for fecundity, and by Chi-square test for mating percentage.

**Figure 4:**
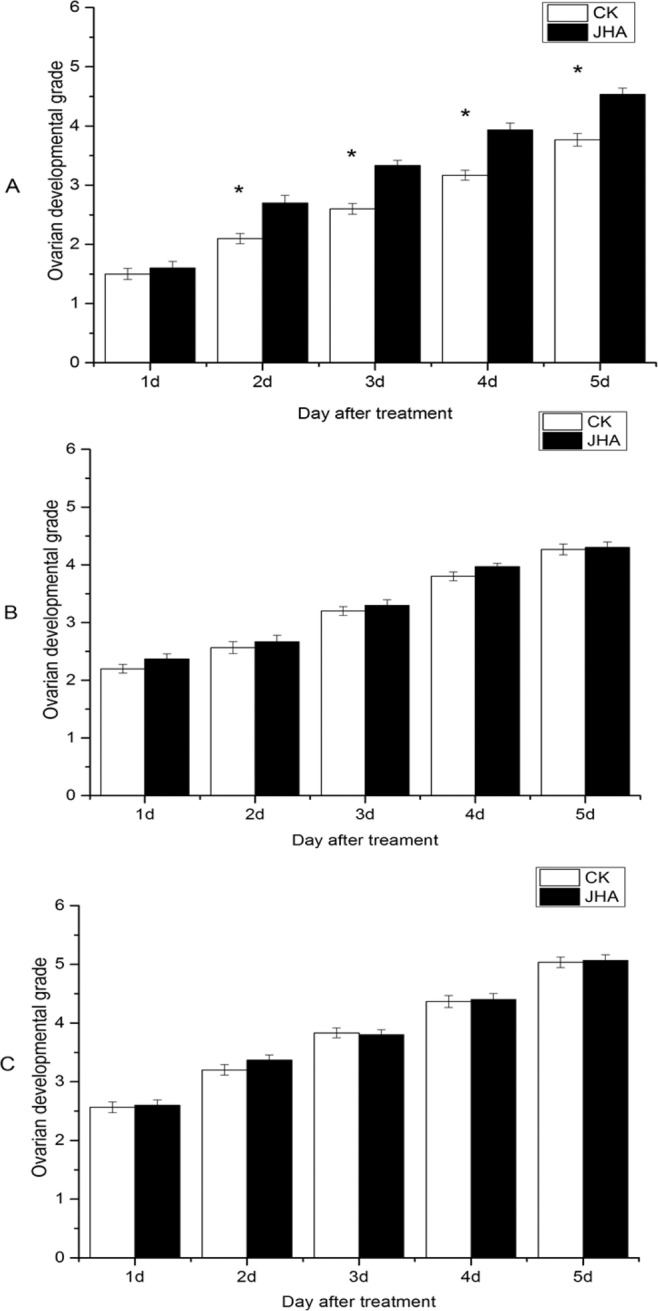
The ovarian developmental grade (mean ± SE) of *Mythimna separata* females on days 1–5 after JHA treatment for the 1-day-old (**A**), 2-day-old (**B**) and 3-day old (**C**) female moths. *indicates significant difference between the JHA treatment and control (CK) at *P* < 0.05 by Student’s t-test.

When the JHA was applied on the 1-day-old females, the moth’s ovary developed faster than these moths without JHA application (Fig. 4A), but when the JHA applied on the 2-day-old or 3-day-old females, their ovary development was as similar as the control without JHA (Fig. 4B,C).

### Effect of JHA on flight

**Figure 5:**
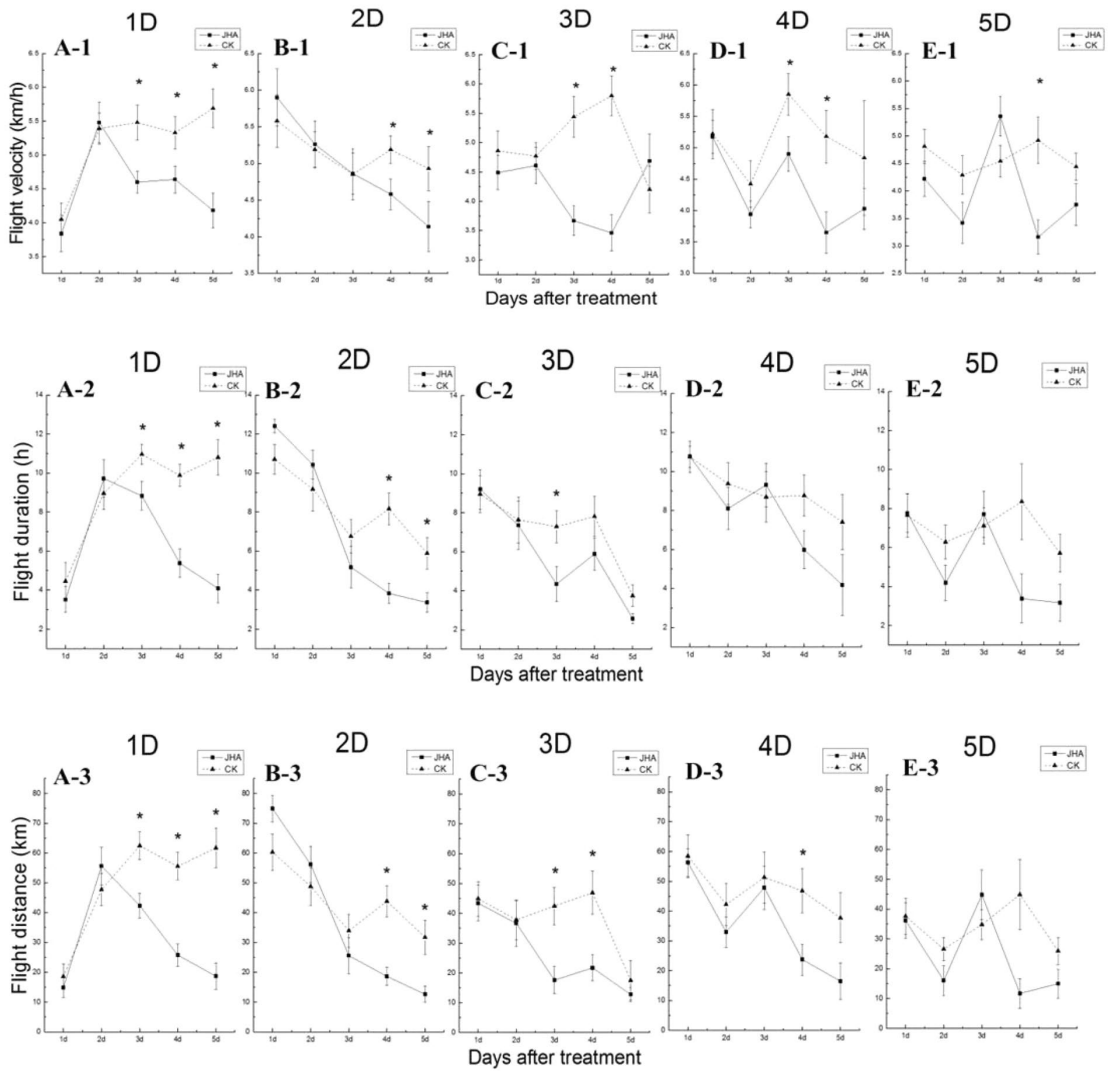
The flight duration, distance, and velocity of females on day one to five after JHA treatment for the one-day-old females (A1-3), two-day-old females (B1-3), three-day-old females (C1-3), four-day-old females (D1-3) and five-day-old females (E1-3). * indicates significant difference between the JHA treatment and control (CK) at *P* < 0.05 by Student’s t-test. On the top of the figures, 1D, 2D, 3D, 4D and 5D indicate the treatment age with JHA/acetone. In the X-axis, 1d, 2d, 3d, 4d, and 5d indicate the day after JHA/acetone treatment.

JHA influenced flight capacity of female *M. separata*. In general, female’s flight capacity decreased when moths were treated with JHA, on the first to fifth day after emergence. However, the later the JHA used, the less suppression on the flight capacity would be found, including the flight velocity, duration and distance (Fig. 5).

**Figure 6:**
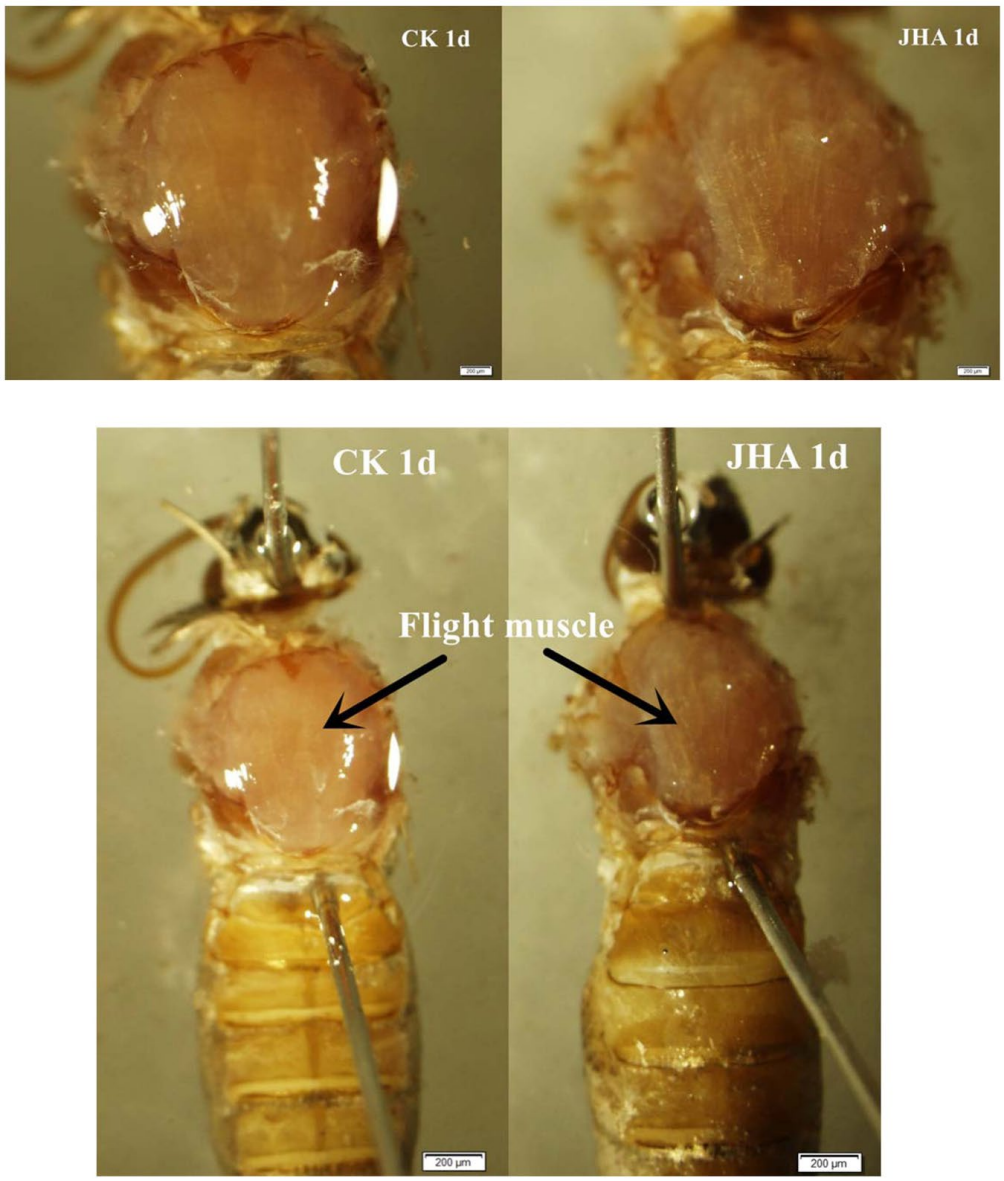
The flight muscle on day 3 after treatment with JHA on 1-day-old female adults of *Mythimna separata*.

**Figure 7:**
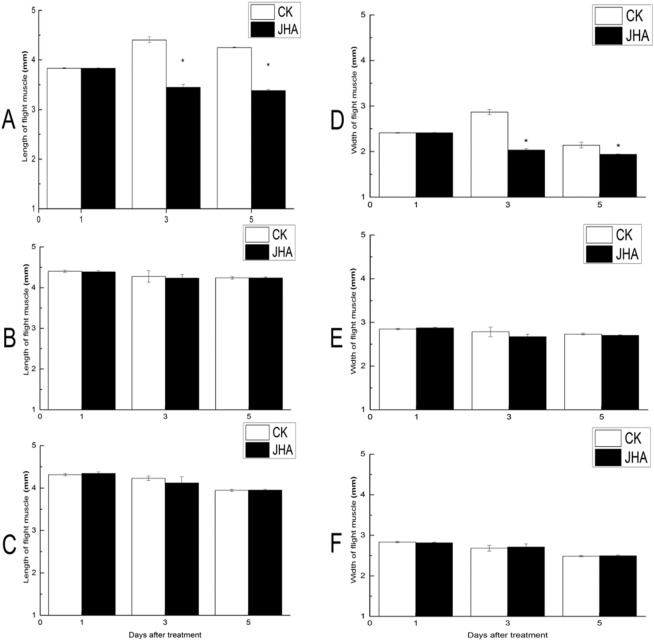
Dorso-longitudinal length and width of flight muscle (mean ± SE) on 1, 3 and 5 days after treatment with JHA on 1-day-old (**A,D**), 2-day-old (**B,E**) and 3-day-old (**C,F**) adult females of *Mythimna separata*. *indicates significant difference between the JHA treatment and their control (CK) at *P* < 0.05 by Student’s t-test.

JHA applied on 1-day-old moths also accelerated the degradation of flight muscle, which became thinner and looser (Fig. 6). Flight muscle size, the length and width of the dorso-longitudinal muscle, significantly decreased at the third (length: *t*_*1, 29*_ = -10.98, *P* < 0.01; width: *t*_*1, 29*_ = -11.67, *P* < 0.01) and fifth day (length: *t*_*1, 23.93*_ = -41.38, *P* < 0.01; width: *t*_*1, 17.81*_ = -3.21, *P* = 0.005) after the JHA treatment on the 1-day-old adults (Fig. 7A,D). However, when the JHA used in the 2-day-old or 3-day-old adults, the flight muscle sizes were not affected (Fig. 7B,C,E,F).

## Discussion

Our results support the hypothesis that JH is an important endocrine regulator in *M. separata*, which can cause the switch from migrants to residents during the adult stage, via accelerating reproduction and decreasing flight activity. We also found that this kind of regulation only occurred in a short sensitive period, the first day after adult emergence. In *M. separata*, migrants have a longer POP and stronger flight capacity than residents^24,27,33^. The decreased flight capacity of JHA-treated adults may result from direct effects on the metabolism of glycerides^36^.

The presence of a sensitive period to JH in the adult stage of *M. separata* differs notably from other wing-polymorphic insects, in which the JH-sensitive period is restricted to one of several periods of the immature development^11^, but the adult period is not sensitive, because these wing-polymorphic insects cannot change their wing morph once adult wings have developed. Although many studies have shown that JH regulation of reproduction and flight potential was related to JH hemolymph titers^19,20,37–39^, such as control of wing-polymorphism in crickets, direct evidence for a sensitive period of JH action in adults has not been demonstrated previously. In this study, we found that JH levels especially in the first day of adulthood also had the potential to regulate migration of *M. separata*.

In all cases studied thus far, an environmental stimulus alters the endocrine mechanism of metamorphosis by altering either the pattern of hormone secretion or the pattern of hormone sensitivity in different tissues^4,40^. As already noted, there are critical JH-sensitive periods during which the JH level can determine the developmental phenotype^4–12^. The “classic model” postulates that the JH titer is critical in the sensitive period of the wing-morph determination^12^. During the sensitive period, if the JH titer is above or below a critical threshold, the phenotype will change. An increase of JH titers in young adults is thought to be related with the ovarian growth of the flightless morph, and there may be a JH titer threshold for migration or ovarian development^11^. The temporal and tissue-specific expression of JH receptors plays an important role in metamorphosis and reproduction^4,12^.

In *M. separata*, JH titers in migrant adults, especially JH II, were too low to be detected in the first day after emergence; however, in resident adults, both the JH I and JH II titers were detected at higher levels on the first day after emergence. It seems that low JH titers allow newly emerged migrant adults to be sensitive to environmental and endocrine factors. Our previous study demonstrated that the sensitive period for the shift from migrants to residents by environment factor occurs only on the 1^st^ day post-eclosion^31^. In this study, we confirmed that this sensitive period also existed in the physiology of JH. The application of JHA on day 1 significantly shortened the POP of the migrant *M. separata* adults, but the application of the same dose of JHA on other days did not have this effect. It seems that the sensitivity of adults to JHA is related to adult age, and day 1 after emergence is the most sensitive period to JHA. Except lower JH titer level in the sensitive stage, it is also possible that JH-receptor genes are upregulated in the JH sensitive period^4,12,14^. In *M. separata*, JH-receptor abundance during day 1 after emergence in the target organs, such as the ovary and flight muscles, was higher than that during other stages which are not sensitive to JH (Zhang *et al*., unpublished data). We hypothesize that methoprene (JHA) activates ovary and flight muscles by combining with JH receptors. Therefore, the higher level of JH-receptor in the sensitive period suggests that methoprene may play a regulatory role in determining ovarian development or flight muscle degradation.

Our results are consistent with the previous finding that day 1 after emergence is the environmentally sensitive period for the shifting of a migrant adult to a resident one in *M. separata*^30–32^. Starvation during day 1 after emergence significantly reduced POP, flight capacity, and dry weight of the thoracic dorso-longitudinal muscle of migrant females, while AT gene expression and the JH titer increased^30–32^. At the same time, in the treatment with JHA on day 1 after emergence the PFO was shorter than that of the control with acetone, indicating synchrony of egg-laying which will serve to increase egg and subsequent larval densities. Similar effects have also been reported in beet webworm, rice leaf roller and oriental armyworm moths, in which flight also increased synchrony of oviposition, leading to increased larval densities and even population outbreaks^9,10^. Additionally, we also found that flight on day 1 after emergence in *M. separata* not only accelerated reproduction but also increased JH titer.

Flight capacity and the flight muscle size of adults with JHA treatment on day 1 after emergence decreased significantly, while reproduction increased. The results show that JH plays important roles in determining the transformation of migrants to residents. We propose the following regulatory pathway for this shift: an environmental signal received in the adult sensitive period (first day after adult emergence) induces the brain to secrete AT. Increasing AT levels stimulate the CA to accelerate the synthesis and secretion of JH. The JH titer increases above a threshold, which redirects the migrant to reproduce, resulting in accelerated ovarian development and earlier onset of oviposition. Meanwhile, degradation of adult flight muscles is induced, and the flight capability significantly decreases. The steps in the transformation of migrants to residents reflect the need for, and the manifestation of, an underlying tradeoff in resource allocation from flight to the reproductive system in *M. separata*. Interestingly, the same JH and AT signaling does not induce the same tradeoff between migrants and residents during other adult ages. Therefore, future studies of the regulatory mechanism of the adult sensitive period of phase changes in *M. separata* should include other potential components, especially the JH receptor. In recent years, major progress on the study of JH receptors in insect species has been documented, such as the genes methoprene-tolerant (MET) and germ cell-expressed (GCE), which are resistant to JH^41^. Both receptors can mediate the metamorphic effect of JH and are essential for pre-adult development in *Tribolium castaneum*, *Drosophila melanogaster* and *Aedes aegypti* but exhibit only minor or indirect effects in reproduction in their respective adult stages^41–52^. Detailed understanding of the molecular basis of JH’s action in female reproduction is available for only a few insects. In *T. castaneum*, JH stimulates vitellogenin (Vg) production indirectly via the insulin signaling pathway^52^. In *Locusta migratoria*, the evidence shows that JH regulates reproduction through MET control of vitellogenesis and oocyte maturation^53–57^. JH and MET also regulate reproduction in *Diploptera punctata* and *Cimex lectularius* in which MET RNAi knockdown results in severe reduction of Vg messenger RNA (mRNA) expression^58–60^.

These results open the door to a better mechanistic understanding of JH-modulated developmental pathways. Therefore, it is possible that the higher expression of JH-MET and JH-GCE during the JH-sensitive period in day 1 after emergence of *M. separata* contributes to a high JH titer after injection of JHA, which is favorable for the switching to resident reproduction from migrants. Further studies are required to determine the role of the JH signaling pathway in directing insect migration and reproduction development.

## Material and Methods

### Insects

The *M. separata* colony was collected from the field in Beijing, China. No specific permits were required for this insect pest collection. The experimental colony was reared for two generations in the laboratory before use in experiments. To obtain migrants and residents, newly hatched larvae were reared at densities of 10 and one larvae per 850-ml jar, respectively, provided with ad libitum fresh corn (*Zea mays*) seedlings (approximately 20-30 cm in length) daily, as described in previous studies^31,32^. Before pupation, the larvae were transferred into sterilized soil (approximately 10% water content). After adult emergence, the adults were immediately transferred in male-female pairs to plastic cages (1000 ml) and provided with a 5% honey solution (v/v) (Acacia flower honey, Hundred Flowers Co, China)^30–32^. Strips of folded wax paper were provided as an oviposition substrate and changed daily. The larvae and adults were maintained at 24 ± 1°C and a 70% RH under a 14: 10 (L: D) photoperiod.

### JH titer determination

JH titers, including JH I and JH II, were determined by HPLC on an HPA1100 (Agilent Company), equipped with an Agilent XDB C18 column (4.6 × 250 mm) and a flow-through UV detector, and the JH extraction was eluted using 80% acetonitrile mobile phase (acetonitrile: water = 80: 20) at a flow rate of 0.8 ml/min, with UV detection monitored at 218 nm under which condition JH I, JH II and other non-target peaks in the sample were separated effectively^31^. Under the separation condition of HPLC, the retention time of JHI and JHII standards by HPLC were was about 16.51 and 20.51 min in UV 218 nm chromatogram (Supplementary Fig. 1A,B), respectively. Under this condition, the substances including JH I, JH II and other non-target peaks in JH extractions from females were all separated effectively (Supplementary Fig. 1C). Hemolymph used for JH titers determination was collected from migrant and resident female adults on 1, 2, 3, 4, 5 and 6 days after emergence. For sampling on each day, a single pool of hemolymph was collected by micropipette at approximately 12:00 am and pooled for a total volume of 50 to 120 μl. This approach required approximately 30 adult females, and three replicates were performed for each age of females. The hemolymph samples were immediately stored at -80 °C after collection. The quantification of JH titers was computed by comparison of the peak area with external quantitative standards that were described by Zhang *et al*.^31^.

### Treatment using a juvenile hormone analogue-Methoprene

A juvenile hormone analogue (JHA) methoprene (ZR515, Sigma Chemical Co, USA) was used to test the effects of JH on *M. separata* reproduction. The first experiment was conducted to determine an appropriate concentration of JHA which could effect reproduction when applied topically^17–19,36^. JHA was dissolved in acetone, and five concentrations (0.006, 0.06, 0.60, 6.00 and 60.00 μg/μL) were used as experimental treatments. Adult females were anaesthetized using diethyl ether and 5 μL of each JHA solution dissolved in acetone was applied to the intersegment membrane between the head and prothorax. Control insects were treated with 5 μL acetone applied to the same region. The JHA treatment was carried out at 12:00 am on day 1 after adult emergence, because this age was previously found to be sensitive to environmental factors which induce JH^31,32^. All the adults were kept under the same rearing conditions as described above. The number of replicates of the control, 0.006 μg/μL, 0.06 μg/μL, 0.60 μg/μL, 6.00 μg/μL, and 60.00 μg/μL treatments were 20, 32, 27, 31, 36 and 27 females, respectively.

Following the dose-dependent JHA experiment, we used a dose of 6 μg/μL JHA (as this dose significantly accelerated adult reproduction) for all further experiments to determine the sensitive age of adults to JHA for regulating reproduction and flight. The newly-hatched male and female moths were paired in a plastic cage and reared under the same conditions as noted above. Female moths were selected and treated with 5 μL of 6 μg/μL JHA at 12:00 am on days 1-5 after emergence (1, 2, 3, 4 and 5-day old), named D1-D5 treatments, and their replicates were 29, 29, 21, 21 and 22, respectively. The same age adults, applied with the same volume of acetone, were the controls of each age, and their replicates were 21, 19, 19, 22 and 20, respectively.

### Reproductive parameter determination

The behavioral and physiological criteria used to evaluate the capacity of females to reproduce (namely, the pre-oviposition period (POP), the period of first oviposition (PFO), oviposition period, lifetime fecundity and mating percentage) were defined and measured following the methods developed in our previous studies^9,10,28,32^. Immediately after emergence, male and female moths were paired and fed. After the JHA treatment, the oviposition was observed daily, and the first and last oviposition dates were recorded to determine the POP, PFO and oviposition period. The POP was the duration from adult emergence to the first oviposition, and the oviposition period was the duration between the first and last oviposition. The POP was used to differentiate migrant and resident phenotypes^27,32^. The PFO is defined as the number of days between a female’s POP and the minimal POP among all females in a treatment^9,10^. For example, a mean PFO of 1 day in a treatment group indicates that, on average, all females begin to oviposit within 1 day after the first case of oviposition in the same treatment group. The lower the PFO, the more synchronized is the onset of oviposition in a treatment, and the greater the resulting larval density^9,10^. Lifetime fecundity was used as a reproductive index of migratory status and was calculated as the total of eggs per female from the first to last day of oviposition^31^. At death, the females were dissected and the number of spermatophores in the bursa copulatrix was determined. The times of mating, and the mating percentage of all treated females could be computed using the rate of the number of females with one or more spermatophores to the total treated females.

Ovarian developmental grades were also measured to assess the effect of JHA on the reproductive system. After JHA treatment on 1-, 2-, and 3-day-old moths, the female ovary was dissected in Phosphate Buffered Saline (PBS) under a binocular microscope and the developmental grade of the ovary was evaluated at 1, 2, 3, 4 and 5 days after JHA treatment, respectively^30,33^. Females treated with acetone on the same age were the control. 30 females were examined in each treatment.

### Flight capacity and flight muscle size assessments

Flight tests were conducted using a 48-channel flight mill system, as described in previous studies^10,24,26,27,33,61^. *M. separata* female adults were anesthetized with ether, and the scales on the junction between the metathorax and abdomen were brushed away. A short hollow plastic tether was glued to the dorsal surface of the metathorax using 502 super adhesives (Beijing Chemical Co.). A tethered moth was attached to the arm of a round-about flight mill (150 cm circumference), and a 14 h experimental flight during the dark period (18:00–08:00) was recorded^9,10,18,24^. The tethered flight environment was maintained at a constant temperature of 24 ± 1 °C and 80% ± 10 RH. The flight capacity of adults treated with JHA in one to five days after emergence was examined. The total flight duration, total distance and average speed were automatically recorded by the Jiaduo Insect Fly Information System (Jiaduo, CO., China). The tethered flight was performed for 1 to 5 days after JHA treatment. The sample sizes for one-day-old treated adults were 14, 16, 30, 32, and 22 females for 1, 2, 3, 4, and 5 days after JHA treatment, respectively; the two-day-old adults were 19, 16, 12, 24 and 13 females for 1, 2, 3, 4, and 5 days after JHA treatment, respectively; the three-day-old adults were 16, 16, 17, 11 and 11 females for 1, 2, 3, 4, and 5 days after JHA treatment, respectively; the four-day-old adults were 14, 14, 16, 16 and 6 females for 1, 2, 3, 4, and 5 days after JHA treatment, respectively; the five-day-old adults were 16, 15, 16, 7 and 14 females for 1, 2, 3, 4, and 5 days after JHA treatment, respectively. Females treated with acetone at the same age as each of the 5 JHA treatments were used as controls; the samples sizes were: 1-day-old: 14, 17, 35, 35 and 18 female moths; 2-day-old: 17, 17, 12, 24 and 19; 3-day-old: 18, 16, 17, 12 and 16; samples size-4-day-old: 16, 12, 15, 18 and 13; 5-day-old: 13, 12, 19, 9 and 21.

Flight muscle sizes, including the length and width of dorso-longitudinal muscle, were measured to assess the effect of JHA on flight muscle development. The dorso-longitudinal muscle represents the largest part of the indirect flight muscles of *M. separata*, and it can be easily dissected from the body^62,63^. After application of JHA to the females on day 1, 2 and 3 after emergence, the dorso-longitudinal muscle was removed from the body on a dissecting plate filled with the isotonic insect Ringer saline under a binocular microscope (Olympus, Japan)^64^, and its length and width at days 1, 3 and 5 after JHA treatments were measured (sample sizes: 1-day-old: 17, 14 and 21; 2-day-old: 19, 19 and 21; 3-day-old: 16, 18 and 20). Adults treated with acetone at the same age were the control (sample sizes: 1-day-old: 16, 17 and 18; 2-day-old: 24, 10 and 16; 3-day-old: 18, 14 and 21).

### Data analyses

The basic statistical analyses, such as t-test and ANOVA, were used in this study. In the experiment to test the differences of JH titers between migrants and residents we use two-way ANOVA analysis. Multiple comparisons were tested using Tukey’s test (HSD), when significant differences were found. Differences in the mating percentage between treatments were compared by Chi-squared test. All statistical analyses were performed using SAS 6.03^65^.

## Supplementary information

Supplementary information
