# Supplementary material for: Juvenile hormone regulates the shift from migrants to residents in adult oriental armyworm, Mythimna separata
Source: Sci Rep. 2020 Jul 15;10:11626. doi: 10.1038/s41598-020-66973-z (PMC7363820; doi:10.1038/s41598-020-66973-z)
Supplement: Supplementary file 1 — Supplementary information [file 41598_2020_66973_MOESM1_ESM.docx]

**Juvenile hormone regulates the shift from migrants to residents in adult oriental armyworm, *Mythimna separata***

Lei Zhang^1^, Lili Cheng^1^, Jason W. Chapman^2,3^, Thomas W. Sappington^4^, Juanjuan Liu^1^, Yunxia Cheng^1^, Xingfu Jiang^1*^

^1^ State Key Laboratory for Biology of Plant Diseases and Insect Pests, Institute of Plant Protection, Chinese Academy of Agricultural Sciences, Beijing, 100193, China

^2^ Centre for Ecology and Conservation, and Environment and Sustainability Institute, University of Exeter, Penryn, Cornwall TR10 9FE, United Kingdom

^3^ Department of Entomology, Nanjing Agricultural University, Nanjing, China

^4^ USDA-ARS Corn Insects & Crop Genetics Research Unit, Genetics Laboratory, Iowa State University, Ames, IA 50011, USA

*Correspondence and requests for materials should be addressed to X.J. (Email: [xfjiang@ippcaas.cn](mailto:xfjiang@ippcaas.cn))

Supplementary Figure legends

Supplementary Fig. 1 The retention time of JH I (A) and JH II (B) standards and the sample of JH extraction (C) of female *M. saparata*.

Supplementary Fig. 1

A

B

C
